# Supplementary material for: Neural efficiency in EFL learning and positive psychology
Source: PLoS One. 2025 Feb 26;20(2):e0314730. doi: 10.1371/journal.pone.0314730 (PMC11864557; doi:10.1371/journal.pone.0314730)
Supplement: Attachment — Submitted filename: Review.docx [file pone.0314730.s002.docx]

**Review of the article “PONE-D-24-35352”**

**Title**

Why it shouldn’t be like this: “Enhancing EFL Learning: The Impact of Positive Feedback on Neural Efficiency and Positive Psychology”

**Abstract**

There are no objectives of the study or questions in the abstract. There is very detail about procedure.

So, the procedure should be short and precise.

**Introduction**

Literature review is more detail and too much. I prefer to be short and precise. There are no objectives or research questions in introduction. Introduction should be concluded with a brief statement of the overall aim of the work and a comment about whether that aim was achieved, but it lacks.

**Materials and Methods**

Study methods and materials are not clear for me. I think it should be expressed shortly and precisely. The Materials and Methods section should provide enough detail to allow suitably skilled investigators to fully replicate your study. Specific information and/or protocols for new methods should be included in detail. If materials, methods, and protocols are well established, authors may cite articles where those protocols are described in detail, but the submission should include sufficient information to be understood independent of these references.

**Results, Discussion, Conclusions**

These sections may all be separate, or may be combined to create a mixed Results/Discussion section (commonly labeled “Results and Discussion”) or a mixed Discussion/Conclusions section (commonly labeled “Discussion”). These sections may be further divided into subsections, each with a concise subheading, as appropriate. These sections have no word limit, but the language should be clear and concise. So, this part should part should fill the above requirements.

**Acknowledgments**

I think it is not correctly said. It should be stated correctly.

**References**

It should be written by “Vancouver” style.
