# Supplementary material for: Neural efficiency in EFL learning and positive psychology
Source: PLoS One. 2025 Feb 26;20(2):e0314730. doi: 10.1371/journal.pone.0314730 (PMC11864557; doi:10.1371/journal.pone.0314730)
Supplement: Attachment — Submitted filename: Review report for.docx [file pone.0314730.s003.docx]

Review report for ”**Enhancing EFL Learning: The Impact of Positive Feedback on Positive Psychology**

**and Neural Efficiency**”.

The present study is interesting, the language is good, and the content and analysis match the title. Yet the following points are recommended to amend some issues:

1. Better than attaching the hypotheses to sections 2.2. and 2.3, it is suggested to introduce the hypotheses in a separate section (2.4) titled Hypotheses of the Study.
2. Section three is to be titled ‘Methodology’ because the study’s methodology as a section is not given due weight. I suggest re-working this section where the research design, population, instruments, rationale, and data collection are introduced before elaborating on the three studies.
3. It is highly suggested to situate Table 1 after presenting all three experiments. It is too early to state the studies finding where the researcher does so.
4. Study 1, 2, and 3 are parts of the Methodology section. Therefore, they should appear as sub-sections.
5. There is no justification why the number of participants is not the same in each study. The same applies to the different duration.
6. The three studies lack consistency in the sense that in the first study the researcher introduces the participants as college students whereas in study 2 and 3 they call them EFL learners.
7. The second reference in the References list should be corrected in that the editor (rather than editors) should appear as “In A.C. Michalos (ed.) …..” rather than starting with the last name.
