# Supplementary material for: Neural efficiency in EFL learning and positive psychology
Source: PLoS One. 2025 Feb 26;20(2):e0314730. doi: 10.1371/journal.pone.0314730 (PMC11864557; doi:10.1371/journal.pone.0314730)
Supplement: Attachment — Submitted filename: Rebuttal_letter_.docx [file pone.0314730.s004.docx]

**Thank you for your valuable feedback and suggestions. I have carefully reviewed each of your comments and have addressed all the concerns one by one. Your feedback has been invaluable, and I have made the following changes. I am confident that these revisions have improved the overall quality of our work. Thank you once again for your thorough review and constructive feedback.**

Reviewer 1

1. Title: Why it shouldn’t be like this: “Enhancing EFL Learning: The Impact of Positive Feedback on Neural Efficiency and Positive Psychology”?

***Response:***

Many thanks for the reviewer’s suggestion. After considering Reviewer 1 and other reviewers’ comments and suggestions, I have changed the title to “Neural Efficiency in EFL Learning and Positive Psychology.”

2. Abstract: There are no objectives of the study or questions in the abstract. There is very detail about procedure. So, the procedure should be short and precise.

***Response:***

I am grateful for the reviewer’s suggestion on the abstract, which has been revised accordingly. I hope that this revision meets the reviewer’s expectations.

“This study aims to investigate how teachers’ positive feedback influences learners’ positive psychology (PP) in English as a Foreign Language (EFL) instruction and to examine how PP enhances learning outcomes through the neural efficiency hypothesis. The study employed three neuroscientific and behavioral experiments to address these questions. Study 1, with 78 college-level EFL learners, examined the effect of teachers' positive feedback on learners' PP and found a significant positive impact. Study 2, conducted with 102 EFL learners, tested the neural efficiency hypothesis and revealed that higher PP improved neural efficiency, with positive feedback moderating this effect. Study 3, involving 54 EFL learners, explored the relationship between neural efficiency and learning outcomes, confirming that enhanced neural efficiency leads to better learning performance. Beyond education, the results underscore the broader relevance of positive psychology and neural efficiency in other domains, such as workplace performance, skill acquisition, and personal development, where maintaining cognitive efficiency and well-being is crucial. These findings highlight the importance of positive feedback in fostering PP, improving neural efficiency, and enhancing learning outcomes. The study offers theoretical contributions and practical recommendations for EFL educators to leverage positive feedback and promote effective learning.”

3. Literature review is more detail and too much. I prefer to be short and precise. There are no objectives or research questions in introduction. Introduction should be concluded with a brief statement of the overall aim of the work and a comment about whether that aim was achieved, but it lacks.

***Response:***

I sincerely appreciate the reviewer’s constructive feedback and insightful suggestions. The literature review section has been revised accordingly and included here.

“Moreover, the overall objective of this study is to examine how teachers’ positive feedback influences PP in EFL learners and investigate how PP enhances learning outcomes through the neural efficiency hypothesis. Specifically, it aims to answer the following questions:

1. Does teachers' positive feedback improve learners' PP?
2. How does PP affect learners’ neural efficiency?
3. Does improved neural efficiency lead to better learning outcomes?

The novelty of this research lies in its experimental design, using frontal alpha asymmetry (FAA) of EEG to objectively quantify the impact of positive feedback (compared to no feedback and criticism) on learners’ PP. Furthermore, it empirically tests the neural efficiency hypothesis to explore how PP enhances performance. The results confirmed the study’s aims, demonstrating that positive feedback enhances PP, which improves neural efficiency, ultimately leading to better learning outcomes. These findings provide valuable theoretical insights and offer practical recommendations for EFL educators seeking to improve learners' experiences and outcomes.”

4. Materials and Methods: Study methods and materials are not clear for me. I think it should be expressed shortly and precisely. The Materials and Methods section should provide enough detail to allow suitably skilled investigators to fully replicate your study. Specific information and/or protocols for new methods should be included in detail. If materials, methods, and protocols are well established, authors may cite articles where those protocols are described in detail, but the submission should include sufficient information to be understood independent of these references.

***Response:***

In the Overview of this Research, the following sentences have been added to point out the purpose of the designed tasks.

***“***The tasks mainly focused on fundamental areas where learners with TOEIC scores between 500 and 650 typically need improvement, such as vocabulary expansion, sentence structure, and comprehension of academic texts.” In the Course Content section, the following information was reported to explicitly state how the designed tasks echoed the participants’ current level of proficiency in English.

"Reading passages are chosen carefully to align with participants’ current abilities, with initial texts drawn from general-interest sources and simplified academic content. Over time, the texts become longer and denser, reflecting TOEFL’s requirements for handling academic reading material.”

5. Results, Discussion, Conclusions
   These sections may all be separate or may be combined to create a mixed Results/Discussion section (commonly labeled “Results and Discussion”) or a mixed Discussion/Conclusions section (commonly labeled “Discussion”). These sections may be further divided into subsections, each with a concise subheading, as appropriate. These sections have no word limit, but the language should be clear and concise. So, this part should part should fill the above requirements.

***Response:***

I appreciate and have carefully considered the reviewer’s suggestions regarding the structure and content of the Results, Discussion, and Conclusions sections. However, I believe the current format is appropriate and aligns with academic research standards for the following reasons:

1. Flexibility in Structure: The reviewer's suggestion to allow for separate or combined sections offers flexibility in presenting the research findings. This approach can be tailored to the study's specific nature and clarity of arguments.
2. Clarity and Conciseness: Clear and concise language is essential for effectively communicating research results. This ensures that readers can easily understand the key findings and their implications.
3. Subsection Structure: The suggestion for subsections with concise subheadings helps organize the content logically, making it easier for readers to navigate and understand the different aspects of the research.
4. No Word Limit: The absence of a word limit allows for a thorough presentation of the results, discussion, and conclusions. This ensures the research is adequately represented and all relevant points are addressed.

In conclusion, the current format for the Results, Discussion, and Conclusions sections effectively addresses the reviewer's recommendations while maintaining a balance between flexibility, clarity, and comprehensiveness. I appreciate the reviewer's feedback and believe that the proposed revisions will further enhance the quality and impact of the research.

6. Acknowledgments
   I think it was not correctly said. It should be stated correctly.

***Response:***

The acknowledgment has been rewritten thoroughly. Thank you very much for the kind reminder.

7. References
8. It should be written by “Vancouver” style.

***Response:***

I appreciate the reviewer’s reminder and I have changed the reference style from APA to Vancouver Style.

Reviewer #2:

1. Technical Soundness and Data Support
The manuscript presents a sound piece of research with well-conducted experiments and clear data that support the conclusions. Below are the details for each experiment:

Study 1: The experiment involved 78 college students and tested whether positive feedback enhances EFL learners' PP. The study design (single-factor between-subjects) and the use of ANCOVA to adjust for baseline differences were appropriate. The results show significant effects of positive feedback on PP (F (2) = 22.952, p < .001, η²p = .383). The study's conclusion is well-supported by the data (e.g., Figure 5 and Table 2), showing a clear enhancement of FAA, an indicator of PP, in the experimental group.
Study 2: This study examined neural efficiency using a within-subjects 3×2 factorial design. The authors appropriately used a two-way ANOVA to examine the interaction between PP and positive feedback, finding significant effects on neural efficiency (F (2) = 6.599, p = .002, η²p = .121). This supports the hypothesis that positive feedback enhances neural efficiency, which is detailed with proper statistical reporting (Table 3, Figure 6).
Study 3: This final study explores the relationship between neural efficiency and learning outcomes. Pearson correlation analysis revealed a strong and significant relationship (r = .563, p < .001), supporting the hypothesis that neural efficiency is positively associated with better learning outcomes (Table 4, Figure 7).
Evidence: The statistical evidence provided in Tables 2, 3, and 4, along with corresponding figures, strongly supports the conclusions drawn from the experiments.

Recommendation: The experiments are sound, and the data support the conclusions. However, it may be beneficial to include a deeper discussion of potential confounding variables in the experiment design.

***Responses:***

I sincerely appreciate the editor and reviewers’ constructive feedback and insightful suggestions. The following revisions have been made in the Limitations section.

“While innovative in combining neuroscientific and behavioral methods, the study's experimental design is not without potential confounding variables that could influence the results. One such confound is the individual differences in learners' baseline PP and motivation levels. Some students may naturally have higher levels of psychological well-being or intrinsic motivation, which could affect how they respond to feedback. Additionally, language proficiency and prior exposure to English could vary among participants, influencing their cognitive load and neural efficiency during learning tasks. Cultural differences in how feedback is perceived could also play a role, as learners from certain cultures may respond differently to positive reinforcement.

Another confound could arise from teacher-student rapport; participants with better relationships with instructors may perceive feedback more positively, which affects their performance. Moreover, fatigue or attention levels during EEG sessions could impact neural measurements, as participants with higher fatigue might exhibit reduced neural efficiency, regardless of feedback. To mitigate these potential confounds, the study employed random assignment and controlled for participants’ proficiency levels. However, future research could further address these issues by using larger, more diverse samples and longitudinal designs to monitor changes over time.” (p. 25)

2. Statistical Analysis
The statistical analysis is rigorous and appropriate, with detailed reporting of effect sizes and significance values. The authors use ANCOVA, two-way ANOVA, and Pearson correlation, all of which are correctly applied to their respective datasets.

Study 1 (ANCOVA): The authors properly adjusted for baseline FAA differences, and the results are significant (F (2) = 22.952, p < .001). The use of η²p (partial eta squared) for effect size gives further insight into the strength of the effect (η²p = .383), which is considered a large effect size.

Study 2 (Two-Way ANOVA): The authors used a two-way ANOVA to examine the interaction between feedback and PP on neural efficiency. The interaction term was significant, with η²p = .121, indicating that the feedback and PP interaction had a notable impact on neural efficiency. The assumptions for ANOVA appear to be met, but it would be helpful to include a check for normality or homogeneity of variances.

Study 3 (Pearson Correlation): The correlation between neural efficiency and learning outcomes (r = .563, p < .001) is both statistically significant and strong, indicating a robust relationship.

Evidence: Tables 2, 3, and 4 provide sufficient statistical details, with p-values and effect sizes correctly reported.

Recommendation: Consider adding a check for assumptions (e.g., normality for ANOVA) and ensure this is mentioned in the methods or discussion section for completeness.

***Response:***

I am grateful for the reviewer’s comments and suggestions. The normality for ANOVA has been done and reported in the text as follows: “Moreover, skewness and kurtosis were computed to evaluate the data distribution's normality. The skewness registered at 0.992, fitting within the normality criteria of -2 to +2. Meanwhile, the kurtosis measured at 0.172, aligning with acceptable norms of -7 to +7. These findings suggest that the data distribution closely resembles normality. Put together, it was sound to use ANCOVA for statistical analysis in Study 1.” (pg. 15)

In Study 2, a normality test was also performed, and the report is as follows: “The residuals of the dependent variable show minimal skewness (0.248) and kurtosis (-0.261), which are well within the acceptable thresholds for normality. These results indicate that the residuals are approximately normally distributed, satisfying the normality assumption of ANOVA.” (p. 18)

3. Data Availability
The data availability statement in the manuscript states that data are available upon request from the corresponding author. However, PLOS ONE prefers that the data be fully available without restrictions. The authors should ideally provide the data through a public repository or include it as part of the supporting information.

Evidence: The Data Availability Statement on page 21 indicates that the data can be requested but is not directly available through a repository or as supplementary material.
Recommendation: To fully comply with PLOS ONE’s data policy, I recommend that the authors upload their data to a public repository or include it in the supporting information.

***Response:***

Thanks for the reviewer’s suggestion. The data for this present study have already been uploaded and shared as per request.

4. Presentation and Language. The manuscript is generally well-written, but there are areas where clarity can be improved. For example:
Introduction: While the background is thorough, some sections could benefit from more concise phrasing. For example, in the paragraph explaining positive psychology (page 3), the phrase "It has been asserted that incorporating PP provides a transformative perspective on language education" could be tightened to improve flow.

***Response:***

Thank you for the reviewer’s constructive suggestions and comments. I have carefully revised the introduction in response to the reviewer's suggestion. It is more concise and direct, maintaining the core meaning while enhancing the overall flow of the paragraph. The sentence highlighted above has been changed as follows: “Scholars argue that incorporating PP offers a transformative lens for language education.”

Grammatical Issues: There are minor grammatical issues throughout, such as in Study 2's description: "Participants of the low PP group sat in front of the computer and watch the scene of April's Breakdown of the movie Revolutionary Road," where "watch" should be "watched."

***Response:***

Thank you very much for the feedback. I have hired a professional editing service to check the grammar throughout the manuscript.

Clarity in Statistical Reporting: In Table 2 (Study 1), the authors mention a "η²p" effect size without explaining this term in the text. Although this is a commonly used metric, it would be helpful to briefly clarify it for readers who may not be familiar with this statistic.

***Response:***

I am grateful for the reviewer’s suggestions. The introduction has been thoroughly revised based on the reviewer’s suggestions and the grammatical issues have been double-checked by a professional editing service. As for the effect size of ANCOVA, *η_p_^2^* is used to determine the proportion of variance in a dependent variable that is explained by an independent variable after accounting for other variables in the model.

Evidence: Examples of unclear or awkward phrasing can be found in Study 2 (page 15) and the introduction (page 3).

Recommendation: A light proofreading for grammatical issues and clarity is recommended. Some phrasing can be tightened, and statistical terms should be briefly explained where necessary.

Summary of Recommendations
Technical Soundness: The study is technically sound, but a discussion of potential confounding variables would strengthen the conclusions.
Statistical Rigor: The statistical analysis is appropriate, but it is advisable to mention checks for assumptions (e.g., normality for ANOVA).
Data Availability: The authors should make their data publicly available, either through a repository or as supplementary material, to fully comply with PLOS ONE's data-sharing policy.
Language and Presentation: The manuscript is clear overall, but minor grammatical issues should be addressed, and the phrasing could be made more concise in some sections.

***Response:***

I am very grateful for the reviewer’s constructive and valuable suggestions. The manuscript has been thoroughly checked and revised accordingly.

Reviewer 3

The present study is interesting, the language is good, and the content and analysis match the title. Yet some points are recommended to amend some issues mainly related to the section of Methodology. The review report is attached:

The present study is interesting, the language is good, and the content and analysis match the title. Yet the following points are recommended to amend some issues:

1. Better than attaching the hypotheses to sections 2.2. and 2.3, it is suggested to introduce the hypotheses in a separate section (2.4) titled Hypotheses of the Study.
2. Section three is to be titled ‘Methodology’ because the study’s methodology as a section is not given due weight. I suggest re-working this section where the research design, population, instruments, rationale, and data collection are introduced before elaborating on the three studies.
3. It is highly suggested to situate Table 1 after presenting all three experiments. It is too early to state the studies finding where the researcher does so.
4. Study 1, 2, and 3 are parts of the Methodology section. Therefore, they should appear as sub-sections.
5. There is no justification why the number of participants is not the same in each study. The same applies to the different duration.

***Response:*** Thank you for your careful review of our study. Upon reevaluation, it appears that the numbers of participants were indeed evenly divided between the control and experimental groups in each study. The duration of each experiment was also identical. Your attention to detail is highly appreciated, and I hope this clarification resolves the concern.

In reference to the third point about the table placement: I appreciate your insights regarding the practices in our field. I would like to point out that the approach taken in this study aligns with common practices in certain areas of research, such as marketing. For instance, Biswas, D., Abell, A., & Chacko, R. (2024) and Ringler, C., Sirianni, N. J., Peck, J., & Gustafsson, A. (2024) presented their findings in the table of the third chapter. I hope this clarification addresses your concerns and underscores the relevance of our methodology.

6. The three studies lack consistency in the sense that in the first study the researcher introduces the participants as college students whereas in study 2 and 3, they call them EFL learners.

***Response:*** I appreciate the reviewer's valuable feedback. I have fixed the inconsistency problem by using the term college-level EFL learners.

7. The second reference in the References list should be corrected in that the editor (rather than editors) should appear as “In A.C. Michalos (ed.) …..” rather than starting with the last name.

***Response:*** This has been corrected*.*

Reviewer 4:

Title and Abstract

Title: The title reflects the article’s content, but it might benefit from being more specific about the research’s unique contribution (e.g., neural efficiency in EFL learning and positive psychology).

Abstract: The abstract effectively summarizes the research; however, it could benefit from explicitly mentioning the sample size in each experiment and clarifying the significance of findings beyond the educational setting.

***Response:***

Thank you very much for the reviewer’s constructive suggestions. I have revised these parts based on the reviewer’s suggestions. The title has been changed to “Neural Efficiency in EFL Learning and Positive Psychology,” and the sample sizes are reported in the abstract. Furthermore, the following sentence is also included to clarify the significance of findings beyond the educational setting.

“Beyond education, the results underscore the broader relevance of positive psychology and neural efficiency in other domains, such as workplace performance, skill acquisition, and personal development, where maintaining cognitive efficiency and well-being is crucial.”

2. Introduction

• Strengths: The introduction provides a comprehensive background on Positive Psychology (PP), EFL learning, and the Neural Efficiency Hypothesis. The justification for combining neuroscientific and behavioral methods is clear.
• Weaknesses: The introduction could benefit from a clearer explanation of how this study differs from previous studies on PP and neural efficiency in language learning. It would be useful to elaborate on the practical implications of the findings for educators in different contexts beyond Taiwan.

***Response:***

Thank you for your valuable feedback. I have revised the Practical Implication section based on your constructive suggestions.

“The findings of this study suggest that online EFL instructors can enhance learners' PP by incorporating positive reinforcement strategies and fostering learner autonomy, helping to alleviate students' fear of failure (Câmpean et al., 2024). Improved PP is linked to more efficient neural processing and can positively impact learning outcomes. However, educators need to recognize that positive feedback alone is insufficient to enhance neural efficiency—it must be paired with perceptual processing tasks (e.g., lexical decision or timed reading exercises) that engage students cognitively.

This study highlights that PP plays a pivotal role in learners’ neural efficiency, reinforcing that cognitive and emotional support must be integrated into teaching methods. Supporting Xie and Guo’s (2023) findings, our research underscores the importance of meaningful feedback in shaping learners’ emotional experiences. Constructive and uplifting feedback promotes language acquisition and learners’ well-being, fostering a positive learning environment. By integrating these research-based approaches into teacher training programs, educators can develop more effective pedagogical strategies and provide personalized support to learners. The study's insights can also inform the development of innovative language learning technologies, such as adaptive platforms that offer tailored feedback and support. This research contributes to a deeper understanding of optimizing language learning experiences in the digital age by exploring the broader implications for online learning contexts and feedback mechanisms.

The implications of these findings may extend beyond Taiwan and are relevant to EFL instructors globally, particularly in online and non-immersion settings. Educators can balance positive feedback with structured cognitive challenges to build learners’ confidence while developing their language proficiency. Moreover, these strategies are adaptable to different educational systems, helping teachers create effective learning environments across various cultural contexts.” (pg. 26)

This revision addresses the reviewer’s comments by:

1. Broadening applicability to global EFL contexts, particularly online and non-immersion settings.
2. Clarifying the connection between PP, neural efficiency, and learning outcomes while noting that emotional feedback must be paired with cognitive tasks.
3. Emphasizing practical recommendations that are relevant across educational systems and cultural contexts.

This version offers educators clear takeaways while situating the findings within a broader framework.

3. Literature Review

• Strengths: The literature review is well-rounded, integrating recent research and theoretical frameworks on positive feedback, PP, and neural efficiency.
• Weaknesses: Some of the references are quite general, and a few more specific studies directly linking PP with neural efficiency in EFL contexts would strengthen the argument. Additionally, a clearer distinction between previous findings and the current study’s unique contribution is needed.

***Response:***

I appreciate the reviewer’s suggestion to strengthen our argument by including more specific studies linking *PP* with neural efficiency in EFL contexts. However, one of the challenges I encountered while writing this present research was the need for studies directly addressing this connection. While prior research has explored cognitive efficiency or processing speed in general language learning contexts, a significant gap exists regarding neural efficiency, specifically in English as a Foreign Language (EFL) environments.

To further clarify the relevance of the included references, I have revised the literature review by highlighting research that, while indirectly related, provides valuable theoretical insight into the PP-neural efficiency link. Additionally, I have refined our argument by emphasizing the conceptual alignment between these studies and our focus on EFL learners, thus bridging the gap where direct evidence is currently lacking.

Furthermore, a clearer distinction between prior findings and the current study’s unique contribution is essential. As noted in the manuscript, this study is the first to investigate the relationship between PP and neural efficiency in the specific context of EFL learners. Thus, it constitutes a novel contribution to the field. While previous studies have examined neural mechanisms in bilingualism and L2 learning, no prior research has systematically explored how PP correlates with neural efficiency, specifically among EFL learners.

I hope these revisions address the reviewer’s concerns, and I am grateful for the opportunity to strengthen the manuscript. Thank you again for your insightful comments.

4. Methodology

• Strengths: The three experiments are clearly described, with a solid justification for the use of EEG and behavioral measures. The experimental design is robust, especially the random assignment of participants and the use of control and experimental groups.
• Weaknesses: The methodology could provide more details on how the tasks were adapted to the proficiency levels of the participants, especially given the inclusion of TOEIC scores as a criterion. It would also help to include more information on potential confounding factors and how they were controlled.

***Responses:***

I appreciate the reviewer’s valuable comments and suggestions. Based on these suggestions and comments, I have revised the mentioned sections: “Reading passages are chosen carefully to align with participants’ current abilities, with initial texts drawn from general-interest sources and simplified academic content. Over time, the texts become longer and denser, reflecting TOEFL’s requirements for handling academic reading material.”

As for the cofounding effects issue “Potential cofounding effects might exist because some learners may perform better on reading or listening tasks related to familiar topics. To mitigate this, the course materials cover a broad range of subjects from humanities, sciences, and social studies, ensuring that all participants are equally challenged. Moreover, proficiency development is influenced by individual motivation and physical well-being. The course schedule is designed to balance intensity and rest, with alternating task types to sustain engagement and prevent burnout.”

5. Results
• Strengths: The statistical analysis is thorough and appropriate for the research questions. The use of ANCOVA and two-way ANOVA tests is justified, and the results are clearly presented with tables and figures.
• Weaknesses: While the findings are significant, the discussion could be expanded to explain the practical significance of the effect sizes (e.g., how much PP improvement translates into real-world language learning outcomes). The interpretation of EEG results, particularly the alpha band, should be linked more explicitly to practical applications in language learning.

***Response:***
Thank you for your valuable feedback. We appreciate your insightful suggestions regarding the need for more discussion on the practical significance of effect sizes and the interpretation of EEG results, especially the alpha band. Below are our responses and our revisions to address these points. In response to your comment, we have expanded the Discussion section to explain better how positive psychology (PP) improvements, as reflected in the effect sizes, translate into real-world language learning outcomes. Specifically:

- Moderate effect sizes observed in the impact of PP interventions (e.g., positive feedback and fostering autonomy) indicate meaningful practical improvements. For example, even small improvements in learners’ emotional well-being can increase motivation and persistence, essential for long-term success in language learning. Learners experiencing enhanced well-being are likelier to engage with challenging tasks and maintain focus during complex activities, such as preparing for high-stakes exams like TOEFL or IELTS.
- The positive psychological gains observed in this study—such as reduced anxiety, increased confidence, and greater emotional resilience—contribute to learners' ability to communicate effectively in real-world scenarios, such as presentations, interviews, or daily conversations in English.
- This finding supports the integration of PP strategies, such as consistent positive reinforcement, goal-setting, and autonomy support, into language instruction to boost learners’ emotional engagement and motivation over time, leading to tangible improvements in their language proficiency.

These expanded points highlight how the effect sizes observed in our study reflect meaningful, practical outcomes for both learners and educators, ensuring the findings are applicable beyond the statistical context. I have also revised the Discussion section to link the EEG alpha band activity findings more explicitly with practical language learning applications.

- Alpha band suppression during task performance, observed in learners with higher emotional well-being, reflects reduced cognitive effort and improved neural efficiency. This suggests that when learners experience positive emotions, they are more likely to engage with language tasks in a relaxed state, allowing cognitive processes to become more automatic and efficient.
- EFL teachers must leverage positive psychology-based strategies to enhance learners' emotional well-being, supporting neural efficiency. For example, uplifting feedback, peer encouragement, and intrinsic motivation through personalized learning goals can reduce learners’ cognitive load, promoting more efficient learning.

6. Discussion

• Strengths: The discussion links the results back to the research questions and hypotheses and provides a clear narrative on how positive feedback enhances PP and neural efficiency.

• Weaknesses: The limitations section mentions the need for ecological validity but does not suggest concrete future directions. It would be helpful to propose specific field-based studies or cross-cultural validations. Moreover, the discussion could benefit from a deeper exploration of how different types of positive feedback (e.g., written vs. verbal) might affect learning outcomes differently.

***Response:***

Many thanks for the reviewer’s comment. I agree with your comment regarding the need to outline more concrete future directions to enhance the ecological validity of our findings. In response, we have revised the Limitations section to suggest the following specific field-based studies and cross-cultural validations:

- Classroom-based studies: Future research should investigate the effects of PP interventions in real-world classroom settings where environmental variables (e.g., peer interaction, task complexity) may influence learners’ emotional and cognitive engagement differently from lab conditions.
- Longitudinal studies: Examining the long-term impact of positive feedback strategies on language proficiency could offer deeper insights into how sustained interventions shape learning outcomes over time.
- Cross-cultural studies: Since learner responses to feedback may vary across different cultural contexts, future research should explore the effectiveness of positive feedback strategies (e.g., autonomy support vs. praise) across different EFL environments, such as in Western and Asian educational systems. This approach would offer cross-cultural validation of the study’s findings and identify culturally sensitive teaching practices.

The reviewer’s insightful suggestion to explore how different types of positive feedback (e.g., written vs. verbal) might affect learning outcomes is greatly appreciated. In response, we have added a discussion on this topic to the Discussion section.

- Verbal feedback (e.g., spoken praise during lessons) may have a more immediate emotional impact. It can foster a connection between teachers and learners and help build confidence in oral communication tasks.
- Written feedback (e.g., comments on assignments or online messages) provides learners a permanent reference to revisit, potentially reinforcing positive emotions over time and supporting self-directed learning. It may be especially beneficial in asynchronous online learning environments, where learners process feedback at their own pace.
- Future research should compare the effectiveness of these feedback types in different learning contexts, such as face-to-face and online classes, to determine which methods are most effective for enhancing emotional well-being and improving language outcomes.

This expanded discussion addresses the different mechanisms and contexts through which feedback operates, helping clarify the practical significance of positive feedback strategies for online and offline EFL instruction.

I hope these revisions address the reviewer’s concerns by proposing actionable future directions and expanding the discussion on the impact of feedback types. Thank you again for your thoughtful suggestions, which have improved our manuscript.

7. Conclusion and Implications
• Strengths: The conclusion appropriately summarizes the main findings and their implications for EFL teaching and learning.
• Weaknesses: The practical implications for educators could be expanded. For instance, how can these findings be integrated into teacher training programs? The broader implications for language learning technology and feedback mechanisms in online learning contexts could be more thoroughly explored.

***Response:***

Thank you for the reviewer's comments on the practical implications of the research. These suggestions provide valuable opportunities to strengthen the study’s impact and relevance. The following passages are added to the practical implication section: “By integrating these research-based approaches into teacher training programs, educators can develop more effective pedagogical strategies and provide personalized support to learners. The study’s insights can also inform the development of innovative language learning technologies, such as adaptive platforms that offer tailored feedback and support. By exploring the broader implications for online learning contexts and feedback mechanisms, this research contributes to a deeper understanding of optimizing language learning experiences in the digital age.”

8. References
• The references are up-to-date and relevant, but there might be room to include more recent studies that specifically address the neural mechanisms underlying PP in language learning.

9. Figures and Tables

• The figures are clear and informative, but some tables (e.g., Table 1 on the overview of studies) might benefit from additional footnotes explaining key terms for readers unfamiliar with the neuroscience aspect.

***Response:***

I am grateful for the reviewer’s suggestions. I have added a note to Table 1 explaining key concepts.

Note: Neural efficiency refers to how well the brain uses energy to process information. A more efficient brain uses less energy to do the same work, leading to better thinking, learning, and memory. Positive psychology includes what makes people thrive and flourish, such as positive emotions and character traits.

10. Ethical Considerations
• The ethical statement is clear, and the study follows proper guidelines. However, it could include more information on how the researchers ensured the emotional well-being of participants who were in the control group (receiving criticism or no feedback).

***Response:***

The criticism in this research indicated that the instructor often pointed out errors while neglecting to acknowledge correct answers. The feedback focused solely on language issues rather than the learners themselves. Students from Confucian-influenced cultures typically experience this type of feedback, as teachers in countries like China tend to provide minimal praise (Gan et al., 2021). As a result, the emotional well-being of participants in the control group—who received either criticism or no feedback—can be assured.

General Comments

• Overall, this article presents an innovative and well-executed study. It makes a valuable contribution to the understanding of how positive feedback and PP enhance neural efficiency in EFL learners. However, there are areas where more practical implications and cross-contextual discussions could enhance the article’s relevance to a broader audience of educators and researchers.

***Response:***

Thank you for your thoughtful feedback and positive evaluation of the article. I appreciate your recognition of the study’s contribution to understanding how positive feedback and positive psychology (PP) enhance neural efficiency in EFL learners. In response to your suggestion, I have enhanced the practical implications and cross-contextual discussion sections. These revisions aim to broaden the article’s relevance to educators and researchers by offering more actionable insights and discussing how the findings apply to various educational contexts. I believe these additions strengthen the manuscript and align with your recommendations.

Thank you again for your valuable suggestions.
